# Sevoflurane-emergence agitation: Effect of supplementary low-dose oral ketamine premedication in preschool children undergoing dental surgery

**DOI:** 10.4103/1658-354X.57878

**Published:** 2009

**Authors:** Ahmed Metwally Khattab, Zeinab Ahmed El-Seify

**Affiliations:** *Ain Shams University, Cairo, Egypt, Doha Clinic Hospital, Department of Anesthesia, Doha, Qatar*

**Keywords:** *Agitation*, *ketamine*, *midazolam*, *pediatric anesthesia*, *premedication*, *sevoflurane*

## Abstract

**Background and Objectives:**

The use of sevoflurane in pediatric anesthesia, which could enable a more rapid emergence and recovery, is complicated by the frequent occurrence of post-anesthesia agitation. This study aims to test the efficacy of adding a low dose of ketamine orally, as a supplement to the midazolam-based oral premedication for reducing sevoflurane-related emergence agitation.

**Materials and Methods:**

Ninety-two preschool children, aged between two and six years, with an American Society of Anesthesiologists physical status I or II, scheduled for elective dental filling and extractions under general anesthesia were included. The patients were allocated into two groups: Group M (46 patients) received oral midazolam 0.5 mg/kg, mixed with ibuprofen 10 mg/kg, while group KM (46 patients) received a similar premedication mixture, in addition to ketamine 2 mg/kg. The acceptance of the drug mixture, the onset of action, and the occurrence of vomiting were monitored over the next 30 minutes. Induction of anesthesia was carried out using sevoflurane 8 Vol% in 100% oxygen via face mask. Anesthesia was maintained with sevoflurane 1.5-2 Vol% in an oxygen-nitrous oxide mixture. After extubation, the standard scoring scale was used for assessing the quality of emergence. Agitation parameters were measured using a five-point scale. Agitated children were managed by giving intravenous increments of fentanyl 1 μg/ kg. The time of hospital discharge allowance was recorded.

**Results:**

Drug palatability, vomiting, and onset of action of premedication; showed no significant differences between both groups. Time of eye opening after discontinuation of sevoflurane showed no significant differences between both groups. Postoperative agitation score and rescue fentanyl consumption were higher in group M than in group KM on admission to the PACU (*P* < 0.01). The time of hospital discharge allowance in group M was longer than in group KM (*P* < 0.05).

**Conclusion:**

Adding a low dose of oral ketamine to midazolam-based oral premedication in preschool children undergoing dental surgery reduced sevoflurane-related emergence agitation without delaying discharge.

## INTRODUCTION

Sevoflurane is widely used in pediatric anesthesia because of fast and well-tolerated inhalational induction, low hepatotoxicity, hemodynamic stability, and rapid emergence from anesthesia.[1] However, the occurrence of emergence agitation in children is the major disadvantage of this volatile anesthetic, with the reported incidence up to 80%.[2]

Emergence agitation (also called emergence delirium) can be defined as a ‘disturbance in a child's awareness of his/her environment with disorientation and perceptual alteration including hypersensitivity to stimuli and hyperactive motor behavior in the immediate post anesthesia period’.[3] It may not only cause injury to the child or to the surgical site, but also lead to the accidental removal of the surgical dressings, IV catheters, and drains. Extra nursing care may often be necessary as well as supplemental sedative and/or analgesic medications. Parents who witness emergence agitation in their child may worry about permanent sequelae.[4]

Rapid emergence and increased pain sensation have been proposed as possible causes of emergence agitation seen with sevoflurane.[5] However anxiolytic premedication and effective analgesia does not necessarily prevent emergence agitation and may even cause delayed recovery and hospital discharge or affect the quality of life of the child after discharge.[6,7]

Various pre-, intra-, and immediate postoperative pharmacological (analgesics, opioids, benzodiazepines, clonidine, and dexmedetomidine) and non-pharmacological techniques were used with the aim of reducing the occurrence of emergence agitation after sevoflurane-based anesthesia, with variable results.[2,8] Oral premedication with midazolam and/or ketamine is widely used in pediatric anesthesia, to reduce emotional trauma, ensure smooth induction, and minimize emergence agitation. However, various dosing regimens were used alone or in combination, with variable efficacies and side effect profiles.[8,9] Only few studies tested the role of intravenous ketamine in reducing emergence agitation after sevoflurane, with no available data regarding its use in small oral doses alone or in combination with oral midazolam.

The aim of the this study was to investigate the effects of adding a low oral dose of ketamine to midazolam-based oral premedication in preschool children undergoing dental surgical procedures, focusing on its effect on sevoflurane-related emergence agitation in comparison to midazolam alone.

## MATERIALS AND METHODS

This was a prospective, randomized, double blind, single center study that was carried out in the Doha Clinic Hospital (75 beds). The child was given a toy by the anesthetist who was going to anesthetize him, and what was going to occur on the morning of surgery was explained to him, but he was blind to the used drug.

The anesthetist who saw the patient preoperatively and anesthetized him was blind to the premedication. Parents were not aware of the premedication components.

**Figure 1:**
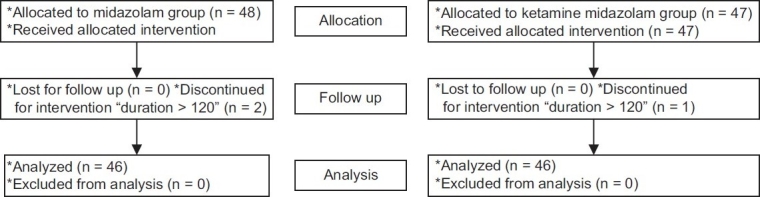
Flow chart of patients through the trial

After receiving approval from the Hospital Ethics Committee and with written consents from the parents, 92 preschool children, aged between two and six years, were scheduled for elective dental filling and extractions under general anesthesia, with the expected duration of surgery being one to two hours. The sample size was determined assuming that the probability of sevoflurane agitation was 40%. We calculated that a sample size of 42 children per treatment group would have at least a 90% power (B error = 0.1) to detect a difference of 30% in the incidence of emergence agitation. Because we expected some exclusions and failures to follow up during the course of the study (which almost did not happen), we increased the number of the sample size to 48 children. Flow chart of patients through the trial [Figure 1].

The children were randomly allowed to participate in this study. Any child who was either ASA I or II was a candidate. Exclusion criteria included, ‘children with developmental problems, cerebral palsy, Down syndrome, inborn errors of metabolism, a history of epileptic fits, body weight less than 10 kg, or greater than 26 kg (Children below age of six years with body weight more than 26 kg were definitely obese with a risk of airway obstruction, along with the abnormal volume of distribution of drugs if given according to their actual body weight). Patients with known allergy to any of the medications used for procedures that lasted less than 30 minutes or longer than two hours, were excluded’. The patients were randomly assigned to receive either midazolam (Group M) or ketamine-midazolam (Group KM) as oral premedication, via computer-generated random numbers.

During the preoperative visit, each enrolled child was asked to choose a sealed envelope with his code number inside. The name, file number, and body weight were recorded on the sealed envelope after been chosen.

On the morning of operation, the study drugs were prepared by a trained nurse (who was not involved in any other part of the study) into identical 5 ml-syringes being sequentially numbered. Labeled syringes were mixed with ibuprofen just before giving to the child. Premedication was given by one of the parents, under the supervision of another nurse.

Group M patients received oral midazolam (Dormicum 5 mg/1 ml-F. Hoffmann-La Roche Ltd, Switzerland) in a dose of 0.5 mg/kg mixed with ibuprofen suspension 10 mg/kg, and Group KM patients received a similar premedication mixture in addition to ketamine (Ketalar™, Pfizer, 50 mg/ml) in a dose of 2 mg/kg. Acceptance of the drug (palatability) [Table 1],[10] onset of action, and the occurrence of vomiting were monitored by the attending nurse over the next 30 minutes. The patient was then transferred to the operating room accompanied by his parent(s). Presence of the parents in the induction room is not routinely permitted in our hospital. Induction of anesthesia was carried out using sevoflurane (Abbott) 8 Vol% in 100% oxygen (6 Ls/min) through a face mask. Intravenous cannulation was allowed for 60 seconds from initiation of mask induction. Atropine 0.02 mg/kg, fentanyl 1μg/kg, and cisatracurium 0.15 mg/kg were given.

**Table 1:** Preoperative behavior scales[10]

Acceptance score (palatability)
Good
Readily accept
Dislike, but accept
Poor
Held down/forced to accept
Refuses to open mouth after tasting

Manual ventilation was started for three minutes, and then intubation with a suitable size, lubricated, and cuffed nasal endotracheal tube was carried out. Anesthesia was maintained using sevoflurane 1.5-2 Vol% in (35:65%) oxygen and nitrous oxide. Ventilation was adjusted to keep end-tidal CO_2_ between 32-40 mmHg. Intravenous paracetamol (Perfalgan^®^ 100 ml vial UPSA France) 15 mg/ kg was infused shortly after induction. All patients were monitored using electrocardiography, pulse oximetry, non-invasive blood pressure/5 min., inspiratory and expiratory N_2_O, O_2_ and sevoflurane, end-tidal CO_2_; and the peak and plateau airway pressures.

At the end of surgery, the anesthetic agents were discontinued (T zero) and replaced with O_2_ 100% (6 Ls/ min). Residual neuromuscular blockade was reversed by neostigmine and atropine. The endotracheal tube (ETT) was removed when the patients were awake. The time from the discontinuation of anesthesia (T zero) till the time of eye opening (defined as the time until the eye opened on verbal command) was recorded.

Agitation parameters were assessed with a five-point emergence agitation scale [Table 2],[8] and measured on admission to the post anesthesia care unit (PACU) and one hour after extubation. Delirium was defined as the agitation score of ≥4 for more than five minutes, despite all calming efforts done by the parents.[1]

**Table 2:** Five-point emergence agitation scale[8]

Score	
1	Obtunded with no response to stimuli
2	Asleep, but responsive to movement and stimuli
3	Awake and appropriately responsive
4	Crying and difficult to console
5	Wild thrashing behavior that requires restraint

In the PACU, parents were allowed to be with their child. Agitated children were managed by giving intravenous increments of fentanyl 1 μg/kg with at least a 10-minute time interval between each dose, during which time the children were monitored for any signs of respiratory depression. All scores and observations were recorded by the anesthesia technician who was unaware of the type of premedication. The time of hospital discharge allowance was recorded (starting from the time of eye opening). Patients were not allowed to go home unless they fulfilled the discharge criteria.[11]

### Statistical methodology

The sample size was determined assuming that the probability of sevoflurane agitation was 40%. We calculated that a sample size of 42 children per treatment group would have at least a 90% power (B error = 0.1) to detect a difference of 30% in the incidence of emergence agitation. Because we expected some exclusions and failure to follow up during the course of the study (which almost did not happen), we increased the number of the sample size to 48 children. Analysis of data was done by IBM computer using SPSS (Statistical program for social science) as follows:-

1. Description of quantitative variables in the forms of mean, standard deviation (SD), and range.
2. Description of qualitative variables in the forms of numbers and percentage (%).

Unpaired *t*-test was used to compare quantitative variables between two independent groups in the parametric data.

Chi-square test was used to compare qualitative variables.

## RESULTS

Ninety-two patients (46 of them were premedicated using midazolam only «Group M» and 46 with midazolam and ketamine «Group KM»), were included in this study from October 2007 to November 2008. Demographic data, duration of anesthesia, and the number of extracted teeth are presented in Table 3 and they showed no evidence of differences between groups. Children of both groups accepted the premedication mixture according to the *Acceptance Score* as shown in Table 4, with no evidence of difference between groups, *P* > 0.05. Vomiting was recorded in one patient in group KM, in whom half of the initial premedication dose was repeated. Although there was no evidence of difference between groups with regard to the onset of action of premedication, clinically the onset was shorter in group KM., as shown in Table 4.

**Table 3:** Patients demographic and operative data (mean ± SD)

	Group (M)	Group (KM)	*P* value
(No.)	46	46	
Age (year)	4.26 ± 1.09	4.1 ± 1.15	0.779
Gender (male/female) (No.)	20/26	28/18	0.672
Body weight (kg)	17.2 ± 3.6	17.4 ± 3.89	0.802
Duration of anesthesia (min.)	87.36 ± 25.06	89.58 ± 26.76	0.753
No. of extractions	2.27 ± 0.98	2.08 ± 1.05	0.330

**Table 4:** Preoperative data and observation

	Group M (%)	Group KM (%)	*P* value
Acceptance of premedication			
Accept (no.-% within group)	36 (78.3)	32 (69.6)	>0.05
Dislike (no.-% within group)	10 (21.7)	14 (30.4)	
Onset of premedication action (min.)	17.82 ± 1.5	16.02 ± 1.2	>0.05

**Figure 2:**
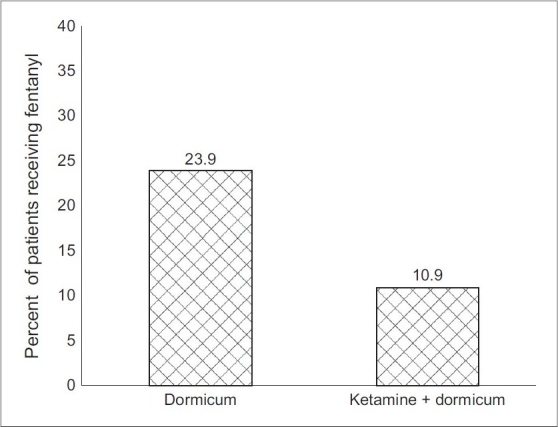
Comparison between both groups with regard to postoperative fentanyl consumption

Time to eye opening, agitation scoring, postoperative fentanyl consumption, and time of hospital discharge allowance are shown in Table 5. Time to eye opening was similar in both groups. On admission to the PACU, 37% (17 out of 46, with 10 of them matching score 5) of the children in group M, developed signs and symptoms of agitation that necessitated giving a rescue dose(s) of fentanyl, in comparison to the agitated children within group KM of 11% (Five out of 46 with three of them matching score 5) *P* < 0.01 [Figure 2]. Fentanyl was given once to all agitated children within group KM, while three of the agitated children in group M needed a second rescue fentanyl dose. Total fentanyl consumption within group M was significantly higher in comparison to group KM (5.08 ± 9.6 vs. 2.021 ± 5.97) *P* < 0.01.

**Table 5:** Comparison between both groups as regards the time of eye opening, agitation score, postoperative fentanyl consumption, and time for hospital discharge allowance

	Group (M) (%)	Group (KM) (%)	*P*value
Time to eye opening (min.)	8.9 ± 1.3	9.6 ± 1.3	*P*> 0.05
Agitation score in (PACU)			
Agitated	17 (37)	5 (11)	**P< 0.01
Non-agitated	29 (63)	41 (89)	
No. of patients with agitation score (5) within group	10 (21.7)	3 (6.5)	*P< 0.05
Postoperative fentanyl consumption (μg), no. of patients and %	5.08 ± 9.6 (11) (23.9)	2.021 ± 5.97 (5) (10.9)	**P< 0.01
Time for hospital discharge allowance (min.)	211.3 ± 21.8	191.7 ± 30.6	*P< 0.05

** = highly significant;

* = significant

The time for hospital discharge of group M (211.± 6 21.8) was longer than group KM (191.7 ± 30.6) *P* < 0.05.

## DISCUSSION

Sevoflurane-related emergence agitation (EA) with a reported incidence of up to 80%, remains a significant post-anesthetic problem that interferes with children's recovery, and challenges the PACU staff in terms of assessment and treatment.[2]

It is, however, usually self-limited (15-30 min.).[5] The exact etiology of sevoflurane-related EA remains unclear. Inadequate pain relief may be the cause of agitation,[7] particularly after short surgical procedures for which peak effects of analgesics may be delayed until the child is completely awake.[12] Intraoperative administration of I.V. ketorolac 1 mg/kg for minor otorhinolaryngological procedures decreases the incidence of EA three to four times, after both halothane and sevoflurane anesthesia.[12] Fentanyl given either I.V. 2.5 μg/kg[13] or intranasal 2 μg/ kg[14] also decreases EA. Similarly; caudal block using clonidine[15] has been seen to be effective.

In our study, we used preemptive ibuprofen (10 mg/ kg), induction I.V. fentanyl 1 μg/kg, and intravenous paracetamol 15 mg/kg to optimize pain management.

On the other hand, post anesthesia agitation has been observed when pain is efficiently treated[7,16] or even when absent.[16]

It has been postulated that rapid awakening after the use of the insoluble anesthetics, such as sevoflurane, may initiate EA.[17]

However, recovery from propofol anesthesia, which is also rapid, is smooth and pleasant in comparison to sevoflurane.[16]

It is becoming increasingly clear that sevoflurane-related EA is probably an intrinsic characteristic of the anesthetic itself, having central nervous effects different from other volatile anesthetics, particularly in younger children.[5] Epileptiform activity has been reported during the use of sevoflurane anesthesia in non-epileptic patients.[18] This could be caused by rapid changes in sevoflurane concentration at the target sites in the brain where the γ-aminobutric acid-ergic properties of sevoflurane would induce changes in the balance between neuronal synaptic inhibition and excitation.[18]

Data on the possible role of premedication in reducing EA were conflicting. Preoperative administration of 0.5 mg/ kg midazolam orally, for 15 minutes before induction, in children undergoing bilateral ear tube insertion reduced the incidence of EA with sevoflurane (from 66.7 to 39.3%),[19] This was almost identical to the 37% incidence of EA recorded within the midazolam-premedicated patients in the current study. Similar studies using oral midazolam premedication in comparison to other drugs failed to show an improved incidence of EA following sevoflurane anesthesia.[12,15]

Ketamine is commercially available as a racemic mixture of R (−) and S (+) enantiomeres. It is a non-competitive N-methyl D-aspartate (NMDA) receptor antagonist that can inhibit the ‘wind up phenomenon’ (central sensitization) in spinal cord neurons. It is this antagonistic effect that accounts for most of the analgesic, amnesic, psychotomimetic, and neuroprotective effects.[20] Two previous studies have been investigated for the efficacy of using intravenous ketamine in small doses, in reducing the incidence of EA following sevoflurane. There are no available studies using oral ketamine to test this point. The results of the current study support the conclusion reached by Kawarguchi *et al*. who used ketamine 1 mg/kg intravenously after induction of sevoflurane anesthesia in children undergoing strabismus surgery.[21] Similarly, small doses of ketamine (0.25 mg/kg) given just before sevoflurane discontinuation in the study carried out by Dalens *et al*. (2006), on children undergoing cerebral magnetic resonance imaging were shown to be effective in ameliorating EA.[22] The discharge time was not prolonged in both studies. Orally administered ketamine has a very high hepatic first pass effect, with only 16% of the dose being bioavailable. Most of the drug is metabolized into norketamine, which may contribute to the analgesic effect.[23]

Oral ketamine premedication avoids the very high peak concentrations produced by I.V. injection, which may be associated with the undesirable side effects.[24]

In animal studies, ketamine was shown to potentiate the release of melatonin, while halothane did the opposite.[25] Melatonin demonstrated beneficial results in children, in attenuating emergence agitation following sevoflurane anesthesia.[26]

One of the limitations of the current study is the absence of a placebo-controlled group. It was initially suggested, but rejected by the Hospital Ethics Committee. There is much debate about the reliability and validity of the tool used to measure emergence agitation in young children. Although the Pediatric Anesthesia Emergence Delirium (PAED) scale, which was developed in 2004,[3] appears to be the most reliable tool for the measurement of EA; yet it was difficult for us to train the observers to apply it in a short time. Therefore we used the simpler and rapidly applicable ‘Five-point scale’. Although the focus of our study was to evaluate EA, one cannot neglect the effects of the used drugs on the induction criteria and the reaction to parents' separation.

## CONCLUSION

Adding a low dose of oral ketamine to midazolam-based oral premedication in preschool children undergoing dental surgery reduces sevoflurane-related emergence agitation, without delaying discharge. The results of this study should open a new concept of ‘multi-modal premedication’.
